# Post-chemotherapy Changes in Bone Marrow in Acute Leukemia With Emphasis on Detection of Residual Disease by Immunohistochemistry

**DOI:** 10.7759/cureus.20175

**Published:** 2021-12-05

**Authors:** Pavithra Ayyanar, Rakhee Kar, Biswajit Dubashi, Debdatta Basu

**Affiliations:** 1 Pathology, All India Institute of Medical Sciences, Bhubaneswar, Bhubaneswar, IND; 2 Pathology, Jawaharlal Institute of Postgraduate Medical Education and Research, Puducherry, IND; 3 Medical Oncology, Jawaharlal Institute of Postgraduate Medical Education and Research, Puducherry, IND

**Keywords:** bone marrow regeneration, dyspoiesis, serous atrophy, stromal changes, trephine biopsy, residual disease, remission, histomorphology of bone marrow, acute leukemia, post-induction chemotherapy

## Abstract

Introduction

In acute leukemia, the leading cause of treatment failure is disease relapse leading to a low level of complete remission and short overall survival. Post-chemotherapy marrow examination gives vital clues regarding treatment response and marrow regeneration.

Aim

We aimed to study the histomorphological changes in post-chemotherapy bone marrow in acute leukemias, monitor residual disease by immunohistochemistry (IHC) on trephine biopsy, and correlate survival status.

Method

This study was a prospective clinical study. A total of 155 post-induction cases (acute myeloid leukemia [AML] - 68 and acute lymphoblastic leukemia [ALL] - 87), from January 2014 to December 2015, were included with a follow-up of 4-28 months. A detailed histomorphology was studied in all cases. IHC was applied in 88 cases of post-induction marrow, which showed morphologic suspicion of an increase in blasts.

Observations

Post-induction marrow was hypercellular in 55.9% of AML and normocellular in 56.3% of ALL. Regenerative hematopoiesis was noted in 37.4% of AML and 88.5% of cases of ALL. Marrow serous atrophy and stromal edema were associated with delayed recovery of counts and their recovery duration ranged from one to five months. Twenty-seven bone marrow aspirates were unsatisfactory, and their trephine biopsies were showed remission in 20 cases and stromal changes in nine cases. In addition, trephine biopsy picked up residual leukemic blasts in four cases in which aspirate showed remission status. Post-induction marrow IHC with scattered positivity for blasts showed sustained remission in 96% cases, and in those with clustered positivity, 28.6% showed residual disease, and 7.2% showed relapse at the end of the study period. The median survival duration was 13, 3, and 12 months for cases with sustained remission, residual disease, and relapse, respectively. There was a statistically significant difference in median survival of patients in the three groups (sustained remission, residual disease, and relapse) (p=0.000).

Conclusion

We conclude that histomorphology augmented by IHC on trephine biopsy gives valuable information regarding post-chemotherapy changes and residual disease status. Bone marrow trephine biopsy is an important tool to assess the remission status of patients with acute leukemia.

## Introduction

In acute leukemia, the leading cause of treatment failure is disease relapse leading to a low level of complete remission and short overall survival [1,2]. Bone marrow examination is performed at the end of induction phase treatment to assess remission status and hematopoietic regeneration [3,4]. In many instances, blasts are very small in number and thereby may be overlooked on light microscopic examination. In addition, hematogones, which are regenerating hematopoietic cells, often resemble the residual blasts morphologically (lymphoblasts), further confounding the true quantification of the residual blasts in acute lymphoblastic leukemia (ALL) [5]. Traditionally, blast count in bone marrow aspirates has been used to assess treatment response. However, post-chemotherapy aspirate smears are often diluted, leading to underestimation of the counts [6]. Trephine biopsies are better in this regard. In addition to the evaluation of the proportion of blasts, trephine biopsies provide information on cellular localization and stromal changes [1,3,5].

The ideal techniques for monitoring residual disease are flow cytometry and polymerase chain reaction (PCR) [7,8]. The newer molecular method, next-generation sequencing (NGS), has been utilized to evaluate minimal residual disease (MRD) in acute leukemia [9]. However, these are expensive, and the facilities may not be available in most centers. In our center, we have attempted to study residual disease by the more readily available technique of immunohistochemistry (IHC) on marrow trephine biopsy. There is a dearth of data regarding this in the literature [1,4,10,11]. We aimed to study the histomorphological changes in post-chemotherapy bone marrow in acute leukemias, monitor residual disease by IHC on trephine biopsy, and correlate survival status in acute leukemia patients.

## Materials and methods

This study was a prospective clinical study conducted in a tertiary care center of South India from January 2014 to April 2016. Inclusion criteria were all acute leukemias, including childhood and adult cases, diagnosed during this period and who had received chemotherapy with a follow-up post-induction marrow. Acute leukemia patients who refused treatment or those who were not on treatment were excluded. During the study period from January 2014 to December 2015, a total of 284 cases (acute myeloid leukemia [AML] - 134 cases and ALL - 150 cases) were newly diagnosed as acute leukemia. Among these cases, post-induction marrow examination was done in 155 cases (AML - 68 cases and ALL - 87 cases) that satisfied our inclusion and exclusion criteria. This study was approved by the Institute Ethics Committee (Human studies), Jawaharlal Institute of Postgraduate Medical Education and Research, Puducherry, and approval number JIP/IEC/2014/8/412.

Study procedure

At the time of the initial presentation, clinical parameters were noted. According to the standard departmental protocol, Leishman and Giemsa stained bone marrow aspirate, and imprint smears were examined. Corresponding biopsies were fixed in AZF solution (acetic acid, zinc, formalin) for 24 hours and decalcification solution (formic acid, formalin, and distilled water) for four hours. Three-micrometer paraffin-embedded sections were stained with hematoxylin and eosin. reticulin stain, Masson trichrome, alcian blue with PAS at pH 2.5, and Perls’ stain were done on morphological suspicion of stromal changes. IHC for CD34 (clone EP88, Pathnsitu, rabbit), CD117 (clone T595, Biogenex, mouse), MPO (polyclonal, Biogenex, rabbit), CD14 (clone MY4, Biogenex, mouse), TdT (clone EP266, Pathnsitu, rabbit), CD10 (clone EP195, Pathnsitu, rabbit), CD79a (clone JCB117, Dako, mouse), CD3 (clone EP41, Pathnsitu, rabbit), and CD20 (clone L26, Biogenex, mouse) were performed according to the cases.

The treatment regime for all AML subtypes (all age group) except acute promyelocytic leukemia (APL) was induction phase - 7+3 regime (Cytarabine100mg/m^2^ for seven days + Daunorubicin 60mg/m^2^ for three days) and consolidation phase with high dose cytarabine 3g/m^2 ^- three cycles, each cycle of which was for a period of 21 days. For APL, induction with ATRA (All trans-retinoic acid) + Daunorubicin and consolidation phase with daunorubicin - for three days - two cycles. The treatment regime for ALL differs according to age and B, T-cell lineage. MCP 841 protocol for pediatric cases of B-ALL, BFM regime for pediatric T-ALL cases, GMALL protocol for adult cases of both B and T-ALL. All these regimes of ALL include the following drugs prednisolone, L-asparginase, vincristine, and daunorubicin.

During follow-up, a complete blood count (CBC) with peripheral smear examination was done to look for the day of the disappearance of blasts and recovery of blood counts. Hematological parameters, including CBC with peripheral smear examination, bone marrow aspirate, and biopsy of the post-induction phase, were noted. The following parameters were studied; cellularity, trilineage hematopoiesis, dyspoiesis, percentage and localization of blasts (scattered/clustered in biopsy sections), hematogones, and stromal changes (marrow fibrosis, necrosis). Based on the National Comprehensive Cancer Network (NCCN) guidelines version 1.2014 - for ALL [12] and version 2.2014 - for AML [13], the complete remission (CR) criteria were as follows: 

i) <5% blasts in bone marrow (in cases of AML, even a single blast with Auer rod was taken as “not in remission”),

ii) Evidence of normal erythropoiesis, granulopoiesis, and megakaryopoiesis,

 iii) Absolute neutrophil count (ANC) > 1×10^9^/ L,

 iv) Platelet count > 100×10^9^/ L. 

Patients with <5% blasts but had incomplete recovery of peripheral blood counts were also categorized as “in remission” for further analysis. 

Relevant immunophenotyping (ICC/ IHC) markers were used in post-induction marrow, which showed the increased blasts on morphology. IHC interpretation of post-induction marrow is as follows. Scattered positivity of blasts, which was less than 5% proportion of marrow cells, and clustered positivity, which showed occasional, small clusters (two to three cells/cluster), which were less than 5% proportion of marrow cells, and these both interpretations were taken as an indicator of regenerating blasts. And for residual neoplastic blasts, the interpretations were as follows: 1) scattered positivity with 5%-10% proportion of marrow cells, 2) clustered positivity with many (>5), large clusters (three to five cells/cluster) [1].

A further marrow examination was done in some instances with persistent low counts, the reappearance of circulating blasts, or had a clinical suspicion of relapse. But we did not assess the post-chemotherapy changes in these cases except to look for the residual blast.

Follow up of all these patients with CBC and peripheral smear examination to look for sustained remission or early relapse until the end of April 2016 with a range of four to 28 months follow-up. At the end of the study period, cases were divided based on marrow status as follows: a) sustained remission - those who continued to be in remission, b) residual disease - those who showed persistence of blasts, and c) relapse - those who show recurrence of disease after attaining remission.

Statistical analysis

Statistical analysis was done using Statistical Package for the Social Sciences (SPSS) software version 16 (SPSS Inc., Chicago, IL, USA). All the categorical variables were presented as frequency and percentages. A paired t-test or Mann-Whitney U test was used for continuous variables accordingly. The chi-square test and Fisher’s exact test were used for the comparison of categorical variables. Differences were considered significant at a p-value of < 0.05. Kaplan Meier estimate was used to estimate the survival function.

## Results

Demographic and clinical details

AML cases were more commonly seen in the age group of 13-40 years (58.8%) and ALL cases were more commonly seen in the pediatric age group ranging from 0 to 12 years (52.3%). Among the 68 AML cases, 48.5% cases were male patients and 51.5% cases were female patients. Among the 87 ALL cases, 73.6% cases were male patients and 26.4% cases were female patients. The cytogenetic study was available for 20/68 AML cases and 15/87 ALL cases. According to the WHO (2008) classification of AML, the diagnosis includes two cases with t(8;21), one case with t(15;17), one case with t(9;11), one case with trisomy 8, one case with del11q (AML with myelodysplasia-related changes category) and a case of blastic plasmacytoid dendritic cell neoplasm. The remaining cases were grouped in AML-NOS (not otherwise specified) category. Regarding ALL cases according to WHO (2008) classification, only two cases (2.3%) were t(9;22); BCR-ABL1 positive. The remaining cases were B- ALL and T-ALL, NOS.

Post-induction marrow features

During the post-induction period, the day of the disappearance of blasts was noted, and it ranged from four to seven days in both AML and ALL cases. Post-induction marrow examination was done in all cases ranging from day 17 to day 35.

**Figure 1:**
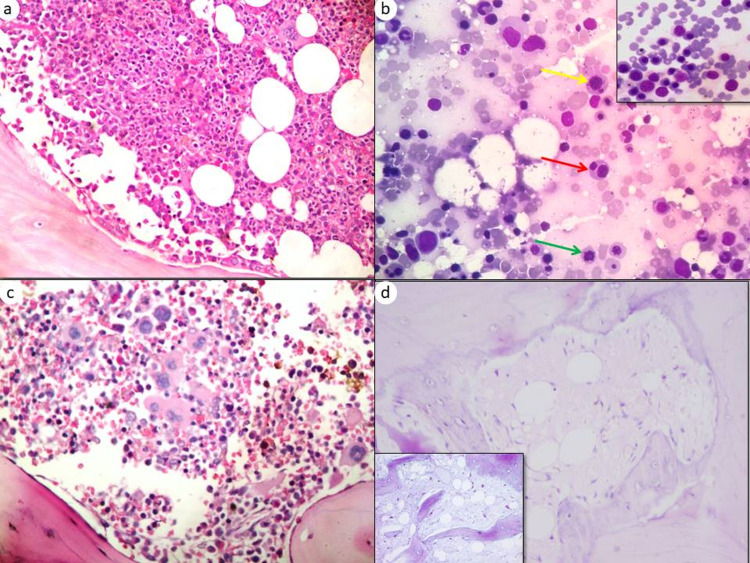
Post-induction marrow histomorphological features (a) Case of AML, post-induction day 28 bone marrow biopsy showing hypercellularity with regenerating hematopoiesis (H&E, 400x). (b) Case of ALL, post-induction day 28 bone marrow aspirate showing dyserythropoiesis - in the form of nuclear budding (yellow arrow), binucleate form (red arrow), and karyorrhexis (green arrows) (Leishman stain, 400x), inset shows megaloblastic erythroid colonies (Leishman stain, 400x). (c) Case of ALL, post-induction day 29 bone marrow biopsy showing dysmegakaryopoiesis- clustering of megakaryocytes and small hypolobated forms (H&E, 400x). (d) Case of ALL, post-induction day 28 bone marrow biopsy showing serous atrophy change (H&E, 400x). Inset highlight the serous atrophy in the AB PAS stain at pH 2.5 (AB PAS at pH 2.5, 400x).

**Figure 2:**
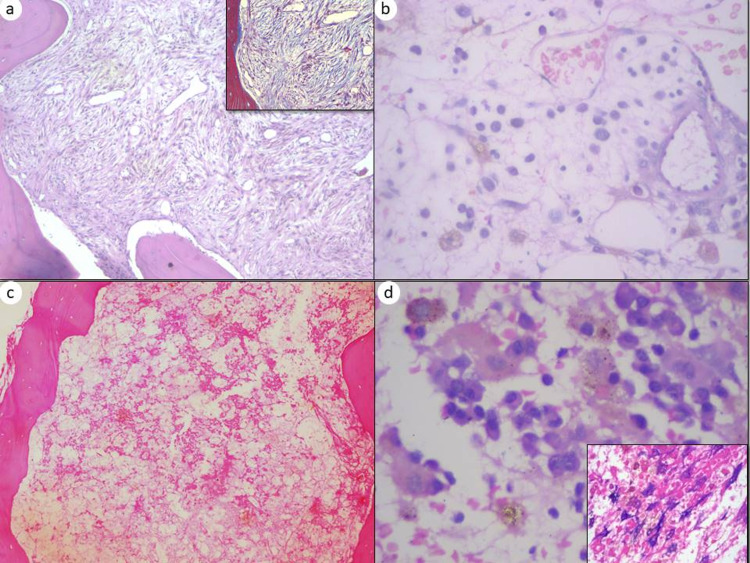
Post-induction bone marrow stromal changes (a) Case of ALL, post-induction day 28 bone marrow biopsy showing marrow fibrosis (H&E, 400x). Insets showing the marrow fibrosis highlighted by Masson trichrome stain (400x). (b) Case of AML post-induction day 19 bone marrow biopsy showing stromal edema (H&E, 1,000x). (c) Case of AML post-induction bone marrow biopsy showing marrow necrosis (H&E, 100x). (d) Case of AML post-induction bone marrow biopsy showing increased hemosiderin-laden macrophages (H&E, 1,000x). Inset showing these macrophages are highlighted by Perls’ stain (Perls’ stain, 400x).

**Figure 3:**
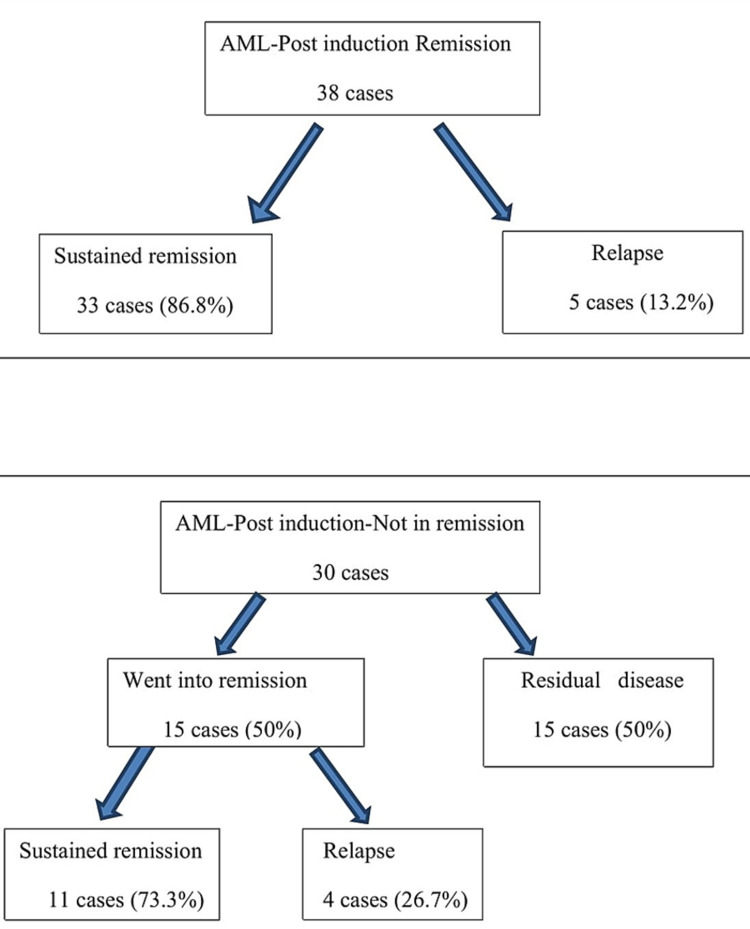
Patient’s disease status at the end of the study period (AML)

**Figure 4:**
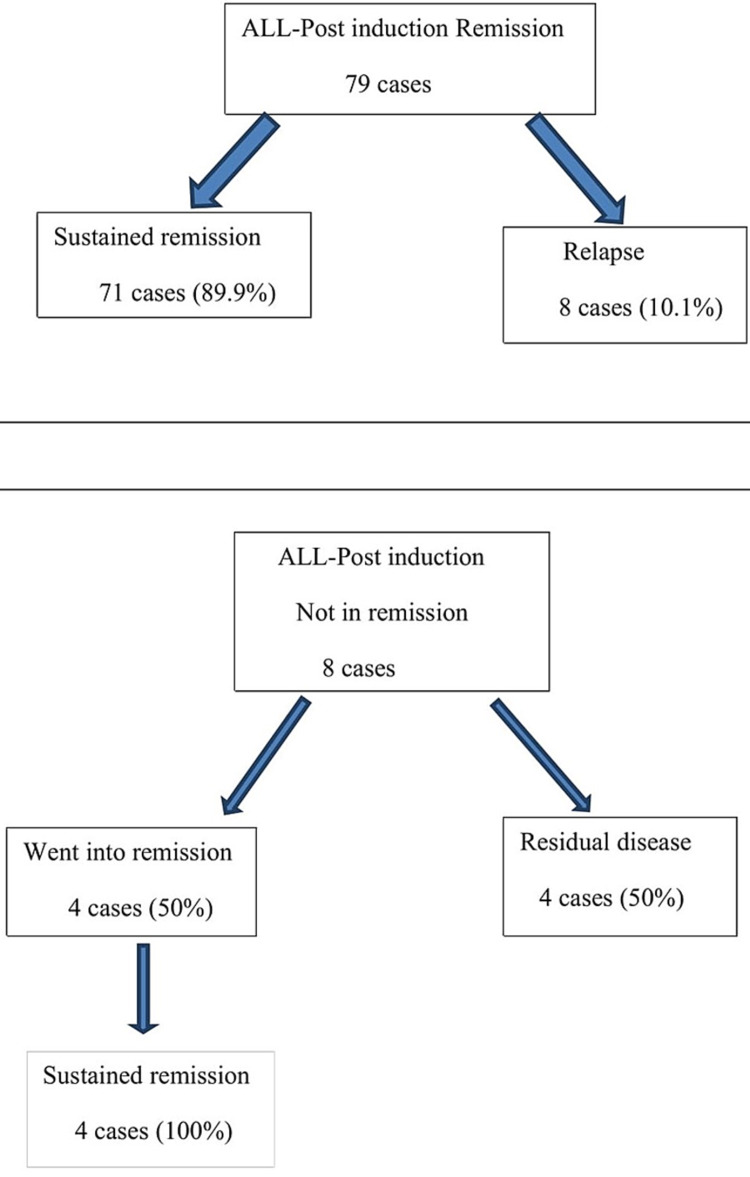
Patient’s disease status at the end of the study period (ALL)

The salient post-induction marrow findings are summarized in Table 1 with images from representative cases illustrated in Figures 1a-1d and Figures 2a-2d. Post-induction marrow status is depicted in Table 2. The patient’s disease status at the end of the study period has been illustrated in Figures 3, 4.

**Table 1:** Post-induction marrow features of all cases Footnotes: *Dyspoiesis was seen in 30 cases out of 68 AML cases and 66 cases out of 87 ALL cases #Stromal changes were seen in 19 cases out of 68 AML cases and 14 cases out of 87 ALL cases ¥Other findings were seen in 33 cases out of 68 AML cases and 30 cases out of 87 ALL cases Abbreviations: AML - Acute myeloid leukemia; ALL - Acutelymphoblastic leukemia

Features	AML, n (%)	ALL, n (%)
Cellularity		
Hypocellular	12 (17.6)	28 (32.2)
Normocellular	18 (26.5)	49 (56.3)
Hypercellular	38 (55.9)	10 (11.5)
Hematopoiesis		
Suppressed	29 (42.6)	10 (11.5)
Regenerative	39 (57.45)	77(88.5)
Dyspoiesis*		
Erythroid lineage	13 (19.1)	41 (47.1)
Myeloid lineage	5 (7.4)	10 (11.5)
Megakaryocytic lineage	12 (17.6)	15 (17.2)
Stromal changes^#^		
Marrow fibrosis	3 (4.4)	2 (2.3)
Serous atrophy	7 (10.3)	7 (8)
Edema	9 (13.2)	3 (3.4)
Necrosis	0	2 (2.3)
Other findings^¥^		
Regenerating fat cells	4 (5.9)	2 (2.3)
Increased macrophages	10 (14.7)	18 (20.7)
Increased plasma cells	11 (16.2)	5 (5.8)
Increased lymphocytes	8 (11.8)	5 (5.8)
Total	68 (100)	87 (100)

**Table 2:** Post-induction status of AML and ALL cases according to response criteria of NCCN guidelines Abbreviations: AML - Acute myeloid leukemia; ALL - Acute lymphoid leukemia; NCCN - National Comprehensive Cancer Network

Post-induction status	Remission			Not in remission, n (%)	Total, n (%)
	Recovery of counts n (%)	Incomplete recovery n (%)	Total, n (%)		
AML	23 (33.8)	15 (22.1)	38 (55.9)	30 (44.1)	68 (100)
ALL	55 (63.2)	24 (27.6)	79 (90.8)	8 (9.2)	87 (100)
Total	75 (48.4)	39 (44.8)	117 (75.5)	38 (24.5)	155 (100)

Bone marrow biopsy versus aspirate

Out of 27 cases where marrow aspirate was inadequate, 20 showed remission on bone marrow biopsy, of which stromal changes including serous atrophy, stromal edema, and increased marrow fibrosis were seen in nine cases. The remaining seven cases showed increased blasts and were “not in remission” on marrow biopsy. Four cases in which post-induction bone marrow aspirate showed remission status, corresponding trephine biopsy picked up residual leukemic blasts, and they were reported as not in remission finally.

Comparison of various parameters among the “remission” and “not in remission” groups

We analyzed the changes in both these groups of acute leukemia, remission, and not in remission, including CBC, peripheral blood smear findings, and bone marrow findings at the time of diagnosis and post-induction day in AML and ALL cases separately. There was no significant difference in CBC and bone marrow parameters. Post-induction day, CBC parameters in AML cases were noted and showed a median (ANC of about 0.415 cells x10^9^/L). The median platelet count was 0.525 cells x10^9^/L in “not in remission” cases, which was a statistically significant difference (p=0.009) compared to remission cases. There was no statistically significant difference between the groups related to post-induction marrow features except regenerative hematopoiesis, which was more commonly seen in “remission” cases compared to “not in remission” ALL cases (p=0.046).

Marrow stromal changes in relation to incomplete recovery of counts at post-induction day

Serous atrophy was seen in 14 cases, of which 10 cases (71.4%) showed incomplete recovery of counts. Similarly, stromal edema was seen in 12 cases, of which six cases (50%) showed incomplete recovery of counts. Marrow necrosis was seen in two cases, of which one case showed incomplete recovery of counts. Marrow fibrosis with incomplete recovery of counts was seen in three cases (3.7%). However, recovery of counts was noted in these cases during follow-up, with a duration ranging from one month to five months.

Interpretation of IHC markers of blasts in post-induction marrow in relation to final marrow status and survival of cases

**Figure 5:**
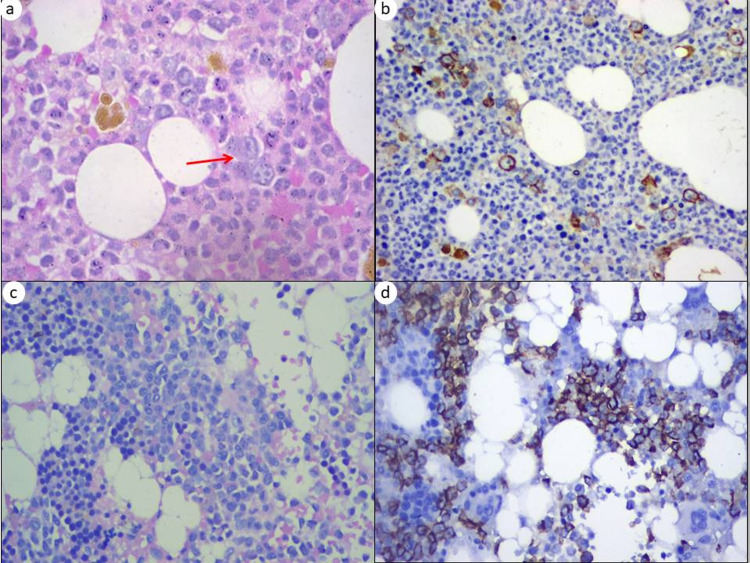
Post-induction marrow immunohistochemical features (a) Post-induction bone marrow biopsy showing scattered and small cluster (two to three cells/cluster) (arrow) of large cells (H&E, 400x). (b) These cells were scattered positivity for CD34 (IHC, 400x). (c) Post-induction bone marrow showing clusters of large cells (H&E, 400x). (d) These cells were clustered positivity for CD34 (IHC, 400x).

Relevant IHC was applied in 88 cases of post-induction marrow, which showed morphologic suspicion of an increase in blasts. Scattered blasts on IHC were seen in 29 cases. Out of 25 cases of <5% scattered positivity, 24 cases (96%) were in sustained remission, and one case (4%) showed relapse at the end of the study period. Scattered positivity with >5%-10% of cells category was seen in four cases, and all were in remission (Table 3, Figures 5a, 5b). We also compared the post-induction marrow IHC-scattered positivity of blasts in relation to the survival status of cases at the end of the study period. Among these 29 scattered positive cases, 21 (84%) cases of <5% positive category cases were alive, and 4 (16%) cases of <5% positive cases had expired at the end of the study period. Scattered positivity with >5%-10% cells was seen in four cases, and all were alive at the end of the study period.

**Table 3:** Post-induction marrow IHC - scattered positivity of blasts in relation to marrow status of cases at the end of the study period Abbreviations: IHC - Immunohistochemistry

Marrow status at the end of the study period	Post-induction IHC - scattered positivity of blasts with proportion of marrow cells		Total, n (%)
	<5% n (%)	>5%-10% n (%)	
Sustained remission	24 (96)	4 (100)	28 (96.6)
Relapse	1 (4)	0	1 (3.4)
Total	25 (100)	4 (100)	29 (100)

Post-induction marrow IHC features in relation to clustered positivity of markers for blasts were seen in 18 cases. Of these, three cases (75%) of clustered positivity with occasional small clusters category were in sustained remission, and one case (25%) showed relapse at the end of the study period. Of 14 cases of clustered positivity with many, large cluster categories, nine cases (64.3%) showed sustained remission, four cases (28.6%) showed residual disease, and one case (7.2%) showed relapse at the end of the study period (Table 4, Figures 5c, 5d). We also compared the post-induction marrow IHC-clustered positivity of blasts in relation to the survival status of cases at the end of the study period. Among these 18 clustered positive cases, 50% cases each of occasional, small cluster positive category cases, and many large clusters positive cases were alive at the end of the study period.

**Table 4:** Post-induction marrow IHC - clustered positivity of blasts in relation to marrow status of cases at the end of the study period

Marrow status at the end of the study period	Post-induction marrow-IHC - clustered positivity of blasts with the size of cluster and proportion of marrow cells		Total cases, n (%)
	Occasional, small clusters, n (%)	Many, large clusters, n (%)	
Sustained remission	3 (75)	9 (64.3)	12 (66.7)
Residual disease	0	4 (28.6)	4 (22.2)
Relapse	1 (25)	1 (7.2)	2 (11.1)
Total cases	4 (100)	14 (100)	18 (100)

Hematogones were noted in two cases (2.5%) of the post-induction marrow of 79 ALL cases based on morphology and IHC. Both cases were in remission. IHC with CD34, TdT, CD10, CD19, CD20 was done and showed a greater number and stronger positivity of cells for mature markers like CD 19, CD20 when compared to immature markers (TdT, CD34). Based on morphology and IHC, these features were indicative of hematogones.

Follow-up

**Figure 6:**
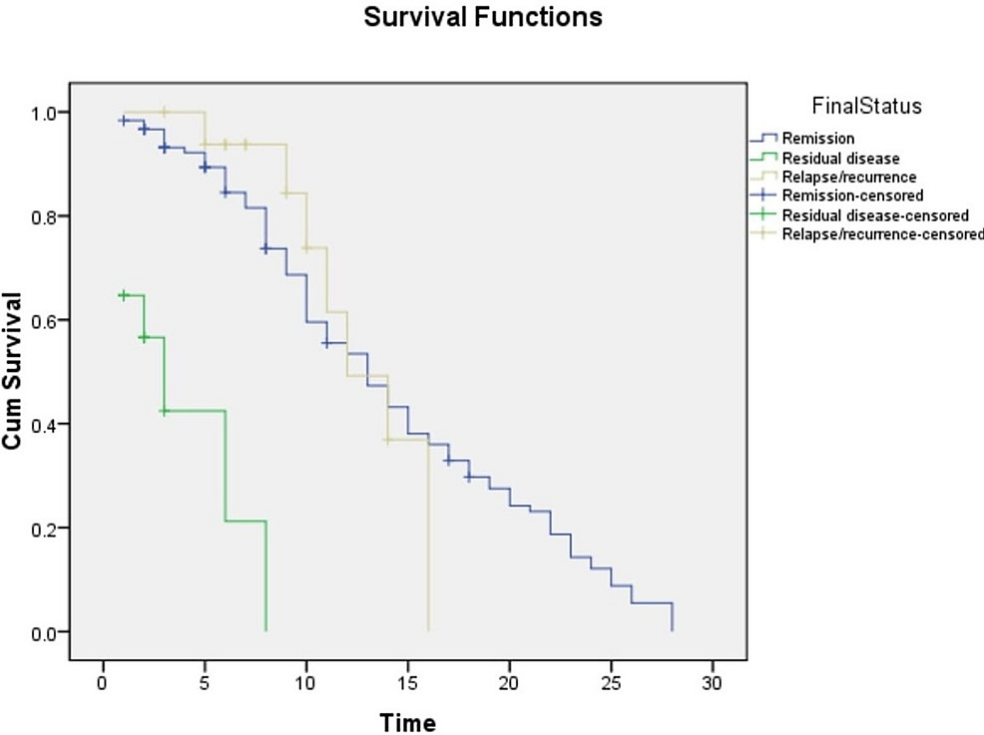
Median survival duration of post-induction categories in relation to final marrow status

In our study, we noted that 85.7% were alive with sustained remission, 90% with residual disease, and 62.5% of relapsed cases had expired. The median survival duration of AML cases and ALL cases were 10 months and 13 months, respectively. We also compared the survival status of cases with the final marrow status. The median survival duration was 13 months for sustained remission cases, three months for the residual disease, and 12 months for relapse cases. Overall median survival duration among the remission versus residual disease cases and residual disease versus relapse category were statistically significant (p<0.001) (Figure 6).

## Discussion

A proper evaluation of bone marrow examination in acute leukemias is necessary to identify relapses, causes, or clues that predict the response during treatment and follow up to improve the sustained remission rate. Despite the current use of flow cytometry and quantitative PCR test for MRD assessment, recently, there were very few studies highlighting the need for post-induction bone marrow examination to monitor residual disease status in addition to the present study. Rathe et al. reported the importance of a complete bone marrow examination (aspirate, imprint, and trephine biopsy) along with IHC in post-induction remission assessment of ALL cases, in addition to the flow cytometry and quantitative PCR, which failed to detect MRD [14]. Similarly, another study (sample size=246) highlighted the role of bone marrow examination (especially bone marrow biopsy) for the morphologic assessment of residual disease in AML [15].

In this study, we have described the histomorphological changes in post-chemotherapy bone marrow in acute leukemias. Post-induction marrow was hypercellular in 55.9% of AML and normocellular in 56.3% of ALL. It is similar to observations by Gerard, who noted normocellular post-induction acute leukemias [16]. In contrast, Islam et al. stated that post-induction bone marrow was predominantly hypocellular in both AML and ALL cases [17]. These different features might be due to variation in the day of the post-induction marrow examination and the chemotherapeutic regime given. Regarding hematopoiesis, our study showed regenerative hematopoiesis in 37.4% of cases of AML and 88.5% of cases of ALL. Similarly, Wilkins et al. also showed regenerative hyperplasia in post-induction marrow at 3-8 weeks of treatment [4]. In our study, dyserythropoiesis was present in 19.1% of AML cases and 47.1% of ALL, noted in the form of nuclear budding, karyorrhexis, binucleate forms multinucleate forms, and megaloblastic colonies. These similar features were noted in 10.7% of acute leukemia cases in the previous study [3]. Dysmegakaryopoiesis was noted in 17.6% of AML cases and 17.2% of ALL cases in the present study. It includes paratrabecular location, clustering of megakaryocytes, and small hypolobated forms. Wilkins et al. noted that megakaryocytic dyspoiesis persisted for long-duration ranging from many weeks to even months [4]. We noted dysmyelopoiesis in 7.4% of AML cases and 11.5% of ALL cases in the form of prominent granules, abnormally lobated forms, and giant forms. Similar features were mentioned in the previous study [3].

In the present study, serous atrophy in post-induction marrow was seen in 10.3% of AML and 8% of ALL; 71.4% of cases of serous atrophy showed incomplete recovery of counts. This finding was similar to Feng's study, which observed thrombocytopenia in the post-chemotherapy AML [18]. Wittels et al. stated that this was a transient finding in four out of 15 acute leukemias [19]. Stromal edema and fibrosis were also noted from the study done by Wilkins et al.; 50% cases of stromal edema and 3.7% cases of increased marrow fibrosis showed incomplete recovery of counts [4]. In our study, we noted one case of myelonecrosis showed an incomplete recovery of counts. A similar finding was observed in previous studies [20,21].

Marrow stromal changes and hypocellularity lead to inadequate and delayed recovery of counts. The proportion of these cases varies amongst different studies depending on the day of the post-induction marrow examination [4,18,20,21]. It also implies that stromal regulations for the normal hematopoiesis need to be adequate in bone marrow after chemotherapy for complete recovery. Recovery of counts was similar to a study by Wilkins et al., who noted this delayed recovery after two weeks of post-induction marrow examination, which was done in the fourth week of chemotherapy [4]. At the end of the study period, marrow status was noted in these cases with stromal changes. We found that the chance of occurrence of residual disease or relapse was lower or unrelated to the stromal findings noted in post-induction marrow. It was similar to the result in a study by Wilkins et al., who reported that relapse was not related to hematopoietic regrowth, including stromal changes [4].

We compared the changes observed between post-induction remission and not in remission categories. However, in the literature search, a similar comparative study was not found. There was no statistically significant difference between the two groups at post-induction day except median ANC value in AML and regenerative hematopoiesis in ALL.

Saini et al. study noted that bone marrow biopsy helped identify residual disease in 3.9% of cases that were not picked up by the bone marrow aspirate sample [15]. Similarly, in the present study, post-induction bone marrow aspirate was diluted and inadequate in 27 cases; however, the corresponding biopsy of seven cases (25.9%) helped to identify the residual disease and the remaining cases (74.1%) were in the CR category. In addition, four patients showed CR on marrow aspirate, and their trephine biopsy highlighted the residual blasts.

In a nine-year follow-up study, Yu et al. reported that clustered precursors in remission cases of post-induction AML (three to five cells/cluster) were the strongest predictor of relapse when compared to the presence of single or scattered precursors. They studied the IHC findings retrospectively in post-induction AML that showed features of complete remission. Further bone marrow biopsies were done after consolidation therapy once every three months in the first year and every six months in the second year. They found that these clustered precursors at the initial biopsy were significantly higher in patients who subsequently relapsed than in those who did not [1]. In the present study, which was done on post-induction marrows with a much shorter duration of 28 months of follow up, 28 out of 29 cases with scattered blasts remained in remission while amongst the 14 cases with clusters of blasts as many as five (35%) showed either residual disease or relapse at the end of the study. Regarding hematogones, histomorphological features (scattered cells which were uniform, highly condensed nuclear chromatin with inconspicuous nucleoli and scant cytoplasm.), and IHC with a greater proportion of cells expressing mature markers like CD19, CD20 when compared with immature markers (CD34, TdT) indicated hematogones. Similar findings were observed by Carulli et al. and Rimsza et al. [22,23].

For survival analysis in acute leukemia, a minimum of five years duration is advised; however, due to time constraints in our study, we assessed follow-up for a period ranging from 4 to 28 months. It was one of the limitations of this study. However, even with this constraint, the overall median survival of remission versus residual disease cases and residual disease versus relapse category were statistically significant (p<0.001).

## Conclusions

Histomorphological changes in terms of cellularity, hematopoiesis, dyspoiesis, and marrow stromal changes in post-chemotherapy marrow were studied in acute leukemia cases. The majority of the cases with stromal changes were although in remission but had incomplete and delayed recovery of blood counts. Post-induction marrow IHC with scattered positivity for blasts showed sustained remission and, in those with clustered positivity, showed residual disease and relapse at the end of the study period. There was a statistically significant difference in the median survival of patients with sustained remission, residual disease, and relapse. We conclude that histomorphology augmented by IHC gives valuable information regarding post-chemotherapy changes and residual disease status.
